# Moyamoya Syndrome in Children With Sickle Cell Disease in Saudi Arabia: A Single-Center Experience

**DOI:** 10.7759/cureus.49039

**Published:** 2023-11-19

**Authors:** Abdalla M Zayed, Sulaiman Al-Muhaimeed, Turki Al-Otaibi, Elsayed Mohammed Ali, Rashid Saleh, Shangrila Joy Ancheta, Fahad Al-Harbi, Khawaja Bilal Waheed, Yasir Albahli, Hamid Alghamdi

**Affiliations:** 1 Pediatrics, King Fahad Military Medical Complex, Dhahran, SAU; 2 Nursing, Prince Sultan Military College of Health Sciences, Dhahran, SAU; 3 Radiology, King Fahad Military Medical Complex, Dhahran, SAU

**Keywords:** children, saudi, moyamoya, stroke, sickle cell disease

## Abstract

Background

Sickle cell disease (SCD) is relatively common in Saudi Arabia. Its neurologic complications such as stroke and Moyamoya syndrome (MMS) can be severe and devastating. Such complications can be minimized by modern investigative tools such as transcranial Doppler (TCD) ultrasound, which is unavailable in many high-risk places. Our aim is to investigate the prevalence and characteristics of these complications in children with SCD in our center where TCD is not available.

Methods

We conducted a retrospective record review of children with SCD admitted to the pediatric ward and visited the pediatric hematology outpatient clinic of King Fahad Military Medical Complex, Dhahran, Saudi Arabia, from January 2010 to December 2021. The target population was children aged six months to 14 years with SCD and a history of stroke or transient ischemic attacks (TIAs). Their magnetic resonance imaging/magnetic resonance angiography (MRI/MRA) of the brain radiographic features were reviewed. A descriptive analysis was used to summarize the demographic characteristics and clinical features of patients with and without MMS.

Results

Twenty-six children (out of 385 with sickle cell anemia, originating mainly from the southwestern and eastern provinces of Saudi Arabia) experienced an overt stroke with an overall prevalence of 6.7%. All patients with stroke were originally from the Southwestern province. Their genotype was SS, and the median age at the onset of the first stroke was six years (IQR: 5.5). The main presenting symptoms were seizures (57.7%), motor weakness (42.3%), headache (15.3%), cranial nerve palsies (11.5%), cognitive deficit (7.6%), and dysphasia (3.8%). The majority of strokes were ischemic (92.3%). MMS was detected in 61.5% and was seen at the onset of the first stroke in all patients with this MRA abnormality. Seven children with moyamoya (43.8%) had recurrent strokes.

Conclusion

In this study, the prevalence of overt stroke is 9% in children with SCD originating from the southwestern region of Saudi Arabia (26/286), and 61.5% of them (16/26) had MMS. It is absent in the children of Eastern origin (99 children). In places lacking TCD facilities, further studies are required to determine if MRA brain screenings of children with SCD may detect MMS before the onset of stroke and help start protective therapy.

## Introduction

Sickle cell disease (SCD) is one of the common hematological disorders in Saudi Arabia, especially in the eastern and southwestern provinces [1]. Cerebrovascular accident (CVA) is a severe complication of SCD [2], which can manifest as silent cerebral ischemia, ischemic/hemorrhagic stroke, or moyamoya syndrome (MMS) [3]. Before the age of 20, about 11% of patients with SCD will have an overt stroke, the most severe type of CVA, unless they receive early therapy [4]. The pathogenesis of stroke involves hemolysis of sickled red blood cells and their abnormal adherence to the vascular endothelium, resulting in changes in the vasomotor tone, a hypercoagulable state, and activation of the endothelial cells [5].

Moyamoya is a pathological condition affecting cerebral vasculature characterized angiographically by progressive stenosis of the supraclenoid carotid artery, anterior cerebral artery (ACA), or middle cerebral artery (MCA) and the development of typical collaterals. This condition may be primary (moyamoya disease) or secondary to a recognized disease (MMS) such as Down syndrome, neurofibromatosis 1, and SCD. Patients with Moyamoya have a lifelong risk of recurrent ischemic strokes leading to poor outcomes [6]. Young children are particularly vulnerable even before the age of two years [7].

For all children (aged two to 16 years) with SCD (HBSS/HBSβ0), transcranial Doppler (TCD) ultrasound should be performed regularly according to the guidelines as it helps early prediction and prevention of stroke [8]. However, TCD ultrasound screening is not available in many parts of the world, especially in developing countries [4]. MRA can detect cerebrovascular disease in very young asymptomatic children [9]. Current guidelines recommend MRI/MRA to detect silent strokes in children of younger school age with HBSS or HBSβ0 thalassemia. However, strokes can occur in children younger than five years, with strokes occurring in children younger than two years of age [10]. Due to the absence of established protocols for screening in this age group, the prevalence of moyamoya in patients with SCD is unknown [11]. In this study, we demonstrate our experience with stroke and MMS in children with SCD at our hospital, where TCD ultrasound was unavailable.

## Materials and methods

Setting 

King Fahad Military Medical Complex, Dhahran, Saudi Arabia, provides medical care to military personnel and their dependents. It is also the referral hospital where patients with SCD are referred from other military health facilities in the eastern region of the country. The vast majority of patients at this hospital belong to two main geographic areas in the Kingdom, Eastern and Southwestern regions. In the pediatric ward and outpatient clinics, children with symptoms and signs of SCD are tested, and the diagnosis is confirmed by hemoglobin (HB) electrophoresis. In this hospital, there are CT and MRI facilities; however, there is no TCD screening.

Policy

In our hospital, patients with SCD presenting with a focal neurologic deficit are immediately evaluated by both a pediatric hematologist and a neurologist. They receive supportive care and are prepared for exchange transfusion as soon as possible. Laboratory investigations include complete blood count, type and cross, HB electrophoresis, coagulation profile, and liver and kidney functions. CT brain scan to rule out cerebral hemorrhage, followed by brain MRI and MRA after the patient is stable. Single volume exchange transfusion is given in the first episode to keep the level of HBS <30%. This is followed by chronic transfusion therapy every three to five weeks to keep the HBS level <30%. This can be achieved by either partial exchange transfusion if HB is >8.5g/dL or simple transfusion if less than that level. These patients are evaluated by the hematologist and neurologist regularly, in addition to the radiological assessment yearly and when indicated.

 Study design

The present research is a retrospective cohort study conducted in our hospital. Pediatric residents reviewed all records of children with SCD (confirmed by HB electrophoresis) and selected those who experienced CVA (TIA, hemorrhagic or ischemic stroke). Included patients were aged six months to 14 years, admitted to the pediatric ward, and attended the pediatric hematology clinic from January 2010 to December 2020. The information was collected in a separate data collection sheet.

Procedure: 

The demographic data were retrieved, including the patient’s age, age at stroke diagnosis, sex, geographic origin, and parental consanguinity. In addition, growth parameters namely, weight, height, and BMI were gathered. All other associated complications of SCD such as acute chest syndrome (ACS), vaso occlusive crisis (VOC), etc., were also reviewed. Data on neurologic disability at the onset and one year after the onset of stroke were collected. These included clinical features such as hemiparesis, visual field deficit, speech deficit, ataxia, decreased level of consciousness, headache, seizures, and deteriorating school performance. The number of strokes and the interval between them were also reported. Baseline laboratory data included HB, mean corpuscular volume (MCV), hematocrit, reticulocyte count, white blood cells (WBCs), platelets, HB electrophoresis, HBF, glucose-6-phosphate dehydrogenase (G6PD) status, and lactate dehydrogenase (LDH). The radiologist reviewed the brain CT and MRI/MRA and confirmed the findings. Stroke laterality, number of infarcts, hemorrhage, Moyamoya Suzuki staging, and any other cerebrovascular abnormalities were studied. Based on the temporal serial variations in the degree of severity of MM, the Suzuki stages [12] are summarized in Table 1.

**Table 1:** Suzuki stages

Grade	Description
Stage 1	Carotid fork narrowing
Stage 2	Moyamoya initiation and dilatation of intracranial main arteries
Stage 3	Moyamoya intensification and anterior cerebral artery (ACA) and middle cerebral artery (MCA) defects
Stage 4	Moyamoya minimization and posterior cerebral artery (PCA) defects
Stage 5	Moyamoya reduction and development of external carotid artery collaterals
Stage 6	The disappearance of moyamoya–associated collaterals

Statistical analysis

In this descriptive study, the patients with stroke were divided into two groups: moyamoya and non-moyamoya groups. Collected data were entered into IBM SPSS version 28 (IBM Corp., Armonk, NY). They are reported as numbers and percentages for categorical variables and mean ± standard deviation (SD) or median (interquartile range) for continuous variables. Prevalence is calculated as the number of people in the sample with the characteristic of interest, divided by the total number of people in the sample.

Ethical approval

This study was approved by the Armed Forces Hospitals Eastern Province - Institutional Review Board (AFHER-IRB) with reference number 2021-019. The study followed good clinical practice and the guidelines of the Declaration of Helsinki.

## Results

Between 2010 and 2021, 385 children with SCD visited the pediatric department inpatient and outpatient clinics. Ninety-nine children were originally from the eastern province, and 286 were from the country’s southwestern region.

Thirty-five children presented with neurological complications of SCD, including overt strokes and transient ischemic attacks (TIAs). Twenty-six children from the series (26/385) had an overt stroke with an overall prevalence of 6.7%. No child from the eastern region had a stroke during this study. All children who experienced an overt stroke were from the southwestern region (26/286), with a prevalence of 9% among southwestern patients. Of these 26 children, two had a hemorrhagic and 24 had an ischemic stroke, and all had the SS genotype. 

Children who had an overt stroke presented with headaches, convulsions, cranial nerve palsies, aphasia, and motor weakness (paralysis/paresis), separately or in different combinations. They had a blood exchange, received supportive care, and were scheduled for chronic transfusion therapy with variable outcomes. One patient was non-adherent to the chronic transfusion therapy. All patients who had seizures received anticonvulsive therapy with complete control. Physio- and occupational therapy contributed to improvement in motor disability. Table 2 shows the distribution of symptoms in our patients (n=26) at presentation and the neurologic sequelae at the one-year follow up.

**Table 2:** Neurologic disability of patients with overt strokes n: number of patients, MM (+): patients with moyamoya, MM (-): patients without moyamoya, Residual disability: after one year follow up

Disability	Cases	Recovered	Residual Disability
1. Seizures [n=15 (57.6%)]:			
MM (+)	10	10	0
MM (-)	5	5	0
2. Paresis [n=11 (42.3%)]			
MM (+)	7	3	4
MM (-)	4	3	1
3. Headache [n=4 (15.3%)]:			
MM (+)	2	2	0
MM (-)	2	2	0
4. Cranial nerve palsies [n=3 (11.5%)]:			
MM (+)	3	1	2
MM (-)	0	0	0
5. Cognitive [n=2 (7.6%)]:			
MM (+)	2	0	2
MM (-)	0	0	0
6. Dysphasia [n=1 (3.8%)]:			
MM (+)	1	0	1
MM (-)	0	0	0

Seizures are the most common presenting symptom of stroke in our patients (n=15 (57.6%)), all of which have been controlled with anticonvulsive therapy. Eleven children (42.3%) presented with motor weakness in their upper and lower limbs. They received physiotherapy with marked improvement. However, five of them continued to have mono paresis (2), hemiparesis (1), and quadriplegia (2). Refractory headache was the main presenting symptom in four patients (15.3%), resolved with supportive care and exchange transfusion. Cranial nerve palsies, mainly facial and bulbar nerves, were observed in three patients (11.5%). Two children (7.6%) experienced ongoing cognitive dysfunction, and one patient (3.8%) had persistent dysphasia.

One patient had an unsuccessful surgical bypass followed by a bone marrow transplant (BMT), and three had BMT without prior bypass surgeries. All bone marrow transplantation procedures were successful.

Nine patients presented with a TIA, including headache, facial palsy, and visual, auditory, or speech abnormalities. Their CT and MRI/MRA scans were unremarkable. Two children had seizures diagnosed as epilepsy after ruling out radiologic abnormalities. One was from the eastern region, and her genotype was SS; the other was from the south, and his genotype was S/B thalassemia. As with patients with overt strokes, children with TIA were scheduled for a chronic transfusion program.

Upon presentation, all children with neurologic manifestations had an emergency CT scan followed by an MRI/MRA confirming the diagnosis. MRA showed 16/26 patients (61.5%) with moyamoya and 10/26 children (38.5%) without this abnormality. Twenty-four (92.3%) patients experienced ischemic strokes, while two patients (7.7%) had hemorrhagic strokes. All moyamoya patients had ischemic strokes. Seventeen patients (65.4%) had single strokes, whereas nine patients (34.6%) experienced recurrent strokes. Of the moyamoya group, nine patients (56.2%) had a single stroke, and seven children (43.8%) had recurrent strokes. Table 3 compares the clinical, laboratory, and radiological characteristics of 16 patients with MMS and 10 patients without MMS who had overt strokes.

**Table 3:** Characteristics of children who presented with overt strokes with and without moyamoya radiologic abnormalities. a Laboratory data: Median (interquartile range [IQR]), Mean (+/-SD). BMI: Body mass index, VOC: Vaso occlusive crisis, ACS: Acute chest syndrome, SSC: Splenic sequestration crisis, Hemolytic C: Hemolytic crisis, HBS: hemoglobin S, HBF: Hemoglobin F, LDH: Lactate dehydrogenase

	Parameter	Stroke n:26	Moyamoya n:16 (61.5%)	No Moyamoya n:10 (38.5%)
Clinical	Age in years: [Median (IQR)]	6 (5.5)	5.5 (4.5)	7 (7.9)
	Sex: Male (%) Female (%)	18 (69.2%) 8 (30.8%)	9 (56.3%) 7 (43.8%)	9 (90%) 1 (10%)
	BMI (kg/m2): Mean( ± SD)	14.7 (+/-3)	15.3 (+/-2.9)	13.6 (+/-3)
	Systolic BP (mm Hg): Mean (+/- SD)	107 (+/-9)	106(+/-8)	109 (+/-11)
	Diastolic BP (mm Hg): Mean (+/- SD)	61 (+/-9)	60 (+/-8)	63 (+/-11)
	Seizures: n (%)	15 (57.7%)	10 (62.5%)	5 (50%)
	Paresis: n (%)	11 (42.3%)	7 (43.8%)	4 (40%)
	Others n (%)	10 (38.5%)	7 (43.8%)	3 (30%)
	VOC n (%)	17 (65.4%)	7 (43.8%)	10 (100%)
	ACS n (%)	7 (26.9%)	4 (25%)	3 (30%)
	SSC n (%)	3 (11.5%)	1 (6.3%)	2 (20%)
	Hemolytic C n (%)	6 (23.1%)	2(12.5%)	4(40%)
	Gall stones n (%)	2 (7.7%)	1 (6.3%)	1 (10%)
	Splenomegaly n (%)	8 (30.8%)	2(12.5%)	6 (60%)
Laboratory^a^	Baseline HB (gm/dL)	7.6 (+/-1)	7.6 (+/-0.8)	7.7 (+/-1.2)
	Hematocrit	22.4 (+/-3.3)	21.8 (+/-2.5)	23.4 (+/-4)
	Reticulocytes%	11 (+/-4.5)	12.6 (+/-4.8)	8.6 (+/-2.8)
	MCV (FL)	81.9 (+/-7.2)	84.2 (+/-6.2)	78.2 (+/-7.3)
	WBCs (x 10³/ul)	14.2 (+/-3.7)	14.7(+/-3.8)	13.6 (+/-3.6)
	Platelets (x 10³/ul)	512 (+/-209)	463 (+/-196)	528 (+/-32)
	HBS (%)	79 (+/-4.9)	78.5 (+/-4.5)	79.9 (+/-5.6)
	HBF (%)	5.7 (+/-3.2)	5.1 (+/-2.3)	6.8 (+/-4.3)
	LDH (U/L)	555 (+/-161)	608 (+/-167)	471 (+/-113)
MRI/MRA	Infarction n (%)	24 (92.3%)	16 (100%)	8 (80%)
	Hemorrhage (%)	2 (7.7%)	0	2 (20%)
	Moyamoya n (%)	16 (61.5%)	16 (100%)	0%
	Number of strokes:			
	Single: n (%)	17 (65.4%)	9 (56.2%)	8 (80%)
	Multiple: n (%)	9 (34.6%)	7 (43.8%)	2(20%)

Table 4 shows the moyamoya changes on the MRA of 16 patients with overt strokes performed shortly after the first stroke and the follow-up one year after.

**Table 4:** MM Suzuki grading in 16 patients with overt strokes *N = Number of patients

MM grade	Description	Number of patients N=16	Single stroke N=9	Recurrent strokes N=7	MRI/MRA follow up after 1 year	
					No change N=3	Worsened N=6
I	Narrowing of the carotid fork	4	4	0	0	2
II	Initiation of the moyamoya	3	2	1	0	2
III	Intensification of the moyamoya	3	1	2	0	1
IV	Minimization of the moyamoya	3	1	2	0	1
V	Reduction of the moyamoya	2	1	1	2	0
VI	Disappearance of the moyamoya	1	0	1	1	0

**Figure 1:**
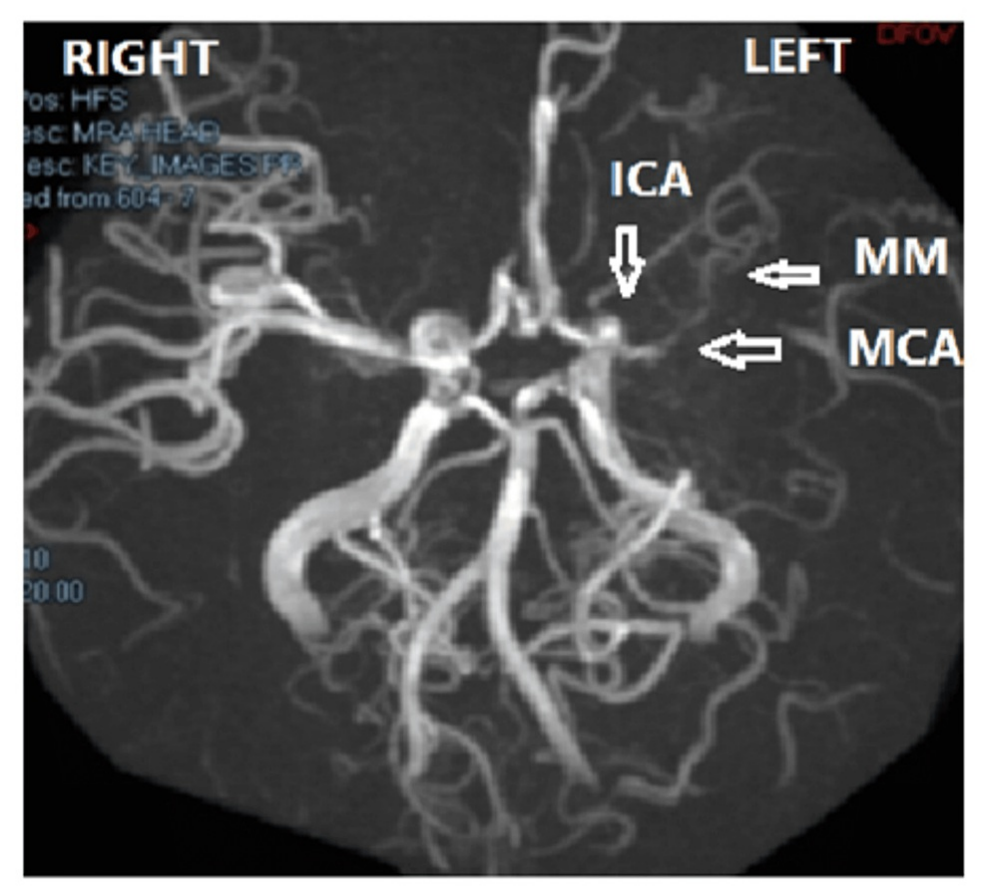
MM stage 2 Obliteration of left supraclenoid ICA (vertical arrow) and MCA (horizontal arrow) with appearance of moyamoya (horizontal arrow).

**Figure 2:**
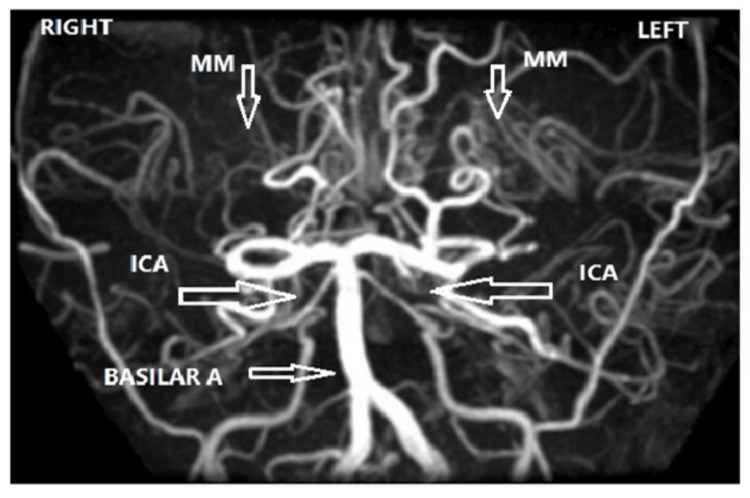
MM stage 3 Bilateral attenuation of ICA and MCA (horizontal arrows) with moyamoya (vertical arrows).

**Figure 3:**
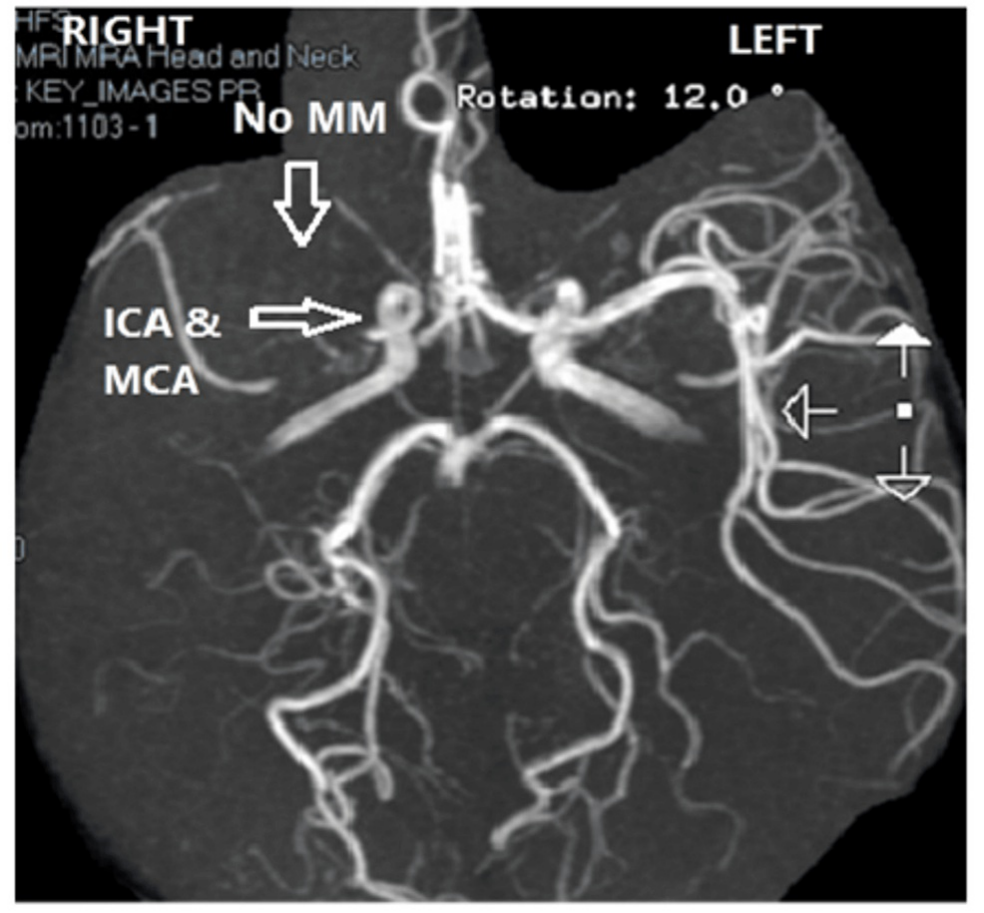
MM stage 6 Non-visualized (obliterated) right supraclenoid ICA and MCA (horizontal arrow) with disappearance of moyamoya vessels (vertical arrow).

The MRI/MRA follow-up scans one year after the first stroke were only available for nine patients, six of whom showed worsening MM. Figures 1-3 are examples of moyamoya changes in the MRA of our patients.

## Discussion

Prevalence of stroke

The prevalence of overt stroke (9%) in children with SCD of southwestern origin and its absence in children of eastern origin is consistent with what was previously reported in Saudi Arabia [13] and could be due to genetic differences between the two groups. There are two different haplotypes of the beta-globin gene in this country [1]: “Arab-Indian” (AI) in eastern and “Benin” in southwestern patients with SCD [14]. Patients from the eastern province are thought to have a milder disease than those in the southwestern region because of the high levels of HBF [1].

Overt strokes occurred exclusively in patients with the SS genotype, and this may be because, in SCD, HBSS leads to increased blood viscosity, further limiting tissue oxygenation [15]. Driscoll et al. [16] reported a prevalence of 7.1% in patients with HbSS and 1.1% in patients with HbSβ0 thalassemia. The risk of stroke in sickle hemoglobin (HbSS) patients has also been reported to be the highest, while it is considered the lowest in sickle β+-thalassemia [7]. Johnson et al. [6] found that among 23 patients with MMS due to SCD, 20 children had genotype SS, two Sβ0, and one SC.

Prevalence of MMS

The prevalence of MMS among Saudi children with SCD is unknown, and no previous studies have yet estimated it. Among our patients with overt strokes, 61.5 % had MMS, consistent with what was previously reported by Dobson et al. [17]. Al-Jefri et al. [18] at King Faisal Specialist Hospital transplanted 14 patients with moyamoya changes with stem cells among 25 children with severe SCD (56%). These patients were referred to the center from different parts of Saudi Arabia. At King Khalid University Hospital, Riyadh, Saleh, et al. [19] described children with strokes between 1992 and 2003, including two children with SCD and others with various diseases with associated MMS. In Sudan, Elmahdi et al. [20] reported MMS in 98% of children who had MRA studies for strokes.

MMS age at diagnosis

The median age of our children at the onset of the first stroke is six years (IQR: 5.5), which is the same as in Uganda [21]. The median age at diagnosis of children with MMS was 5.5 years (IQR: 4.5), consistent with that reported by Johnson et al. [6]. One child had a stroke at 1.4 years of age. She was found to have MMS. She has 3-M syndrome (a rare form of dwarfism). Whether her MM is secondary to SCD, or dwarfism is unknown. However, four other children with SCD had their first stroke associated with MMS before the age of four years. Three out of 29 infants aged seven to 48 months were found to have MRA abnormalities in one study [22]. Since MRI/MRA was performed following a clinically overt stroke, it may be beneficial to screen this group of children for MM changes by performing MRI/MRA before age four in areas where TCD is unavailable.

Stroke and MMS presenting symptoms

The main presenting symptoms of stroke in children with MMS were seizures and motor weakness, followed by other symptoms, including headache, dysphasia, and cranial nerve palsies. Al-Jefri et al. [18] found similar symptoms before BMT in their patients, while in Sudan, Elmahdi et al. [20] reported motor weakness in their entire study population. However, the signs and symptoms of stroke vary due to its location and size variability [23]. Children in our series suffer from other co-morbidities related to SCD, mainly VOC and ACS. These findings are similar to the study conducted in the western region by Halawani et al. [24]. Previous studies considered recurrent ACS a risk factor for ischemic stroke [4]. We still could not demonstrate this because of the small number of our patients. Also, all children in the study without moyamoya had VOC as a co-morbidity, the reason for which is unclear, and further investigation on a larger number of patients is required.

Lab abnormalities in stroke

The median baseline HB in our patients with SCD and moyamoya is 7.6 g/dL (IQR: 0.8), similar to the value in other studies [6], and the difference from the non-moyamoya group is negligible. In several studies, low baseline HB and MCV, leukocytosis, high reticulocyte counts, and LDH levels are considered risk factors for CVA [4]. These abnormalities have been demonstrated in our results and other studies conducted on children with SCD in Saudi Arabia [13,24]. A larger number of patients is required for statistical confirmation.

Moyamoya and stroke occurrence and recurrence

The progressive cerebrovascular stenosis leads to decreased blood flow to the brain, causing cerebral ischemia. The risk of an ischemic event increases with hypoxia, hypotension, hypocarbia, or hyperthermia. Late angiographic stages often correlate with a progressive clinical deterioration [25].

It has been shown that moyamoya can appear before an overt stroke [6], and this phenomenon was reported in a child three months before the stroke [11]. Screening for moyamoya is unavailable at our hospital, and all of our moyamoya patients demonstrated this abnormality on MRA at the time of their first stroke. In a prospective study of 40 patients with overt strokes who received chronic transfusion therapy followed by a series of MRI/MRA, 63% had vasculopathy at the onset of their first stroke [26].

If left untreated, the risk of stroke recurrence increases to approximately 70% but is reduced to 10%-20% with chronic transfusion therapy. In a retrospective study of 137 children treated with chronic transfusion therapy for ischemic stroke at 14 centers, 22% had a recurrent stroke [23]. However, despite optimal blood transfusion, vasculopathy can progress in patients with a history of acute stroke [26].

Among our patients with overt strokes, 34.6% had recurrent strokes despite being on chronic transfusion therapy to keep their HBS below 30%, consistent with Dobson et al. [17]. This could be due to our cohort's relatively high number of patients with MMS. In addition, military people are a moving population, resulting in non-compliance with transfusion programs in some cases, which could also be a contributing factor.

While moyamoya is a risk factor for recurrent stroke [11], two children of the non-Moyamoya group (20%) had a recurrent stroke. In comparison, seven children with MMS (43.8%) suffered recurrent overt strokes with more cerebral damage and more severe residual neurologic disability, consistent with the findings of Elmahdi et al. [23] and Dobson et al. [17]. Sixty-nine percent of patients with ischemic stroke and MMS experienced recurrent ischemic events in Johnson’s 10-year study [27].

Stroke type

Compared to ischemic stroke, which is most common in children, hemorrhagic stroke is more common between the ages of 20-29 years and rarely occurs before or after this range [3]. In our cohort, only two patients (7.7%) had a hemorrhagic stroke, while 24 children (92.3%) had an ischemic stroke. All patients with moyamoya belong to the ischemic group, which is consistent with the findings of Dobson et al. [17], who suggested that the young age of these patients may be the cause.

MMS treatment

Surgical revascularization can prevent stroke by restoring adequate blood flow to the under-perfused areas in the brain [25]. Small studies have demonstrated the safety and efficacy of revascularization procedures in patients with MMS, but randomized controlled trials are still needed to confirm these findings [28]. Due to limited resources, only one of our patients underwent bypass surgery. Unfortunately, he had a new ischemic stroke on the same side of the operation. However, he then underwent a successful stem cell transplant.

Bone marrow transplantation is curative for high-risk children with SCD and moyamoya. The outcome is favorable with stabilization of cerebral vascularity and improvement of symptoms such as headache, convulsions, and hemiparesis [18]. However, this remains limited by the availability of an HLA-matched sibling donor [29]. Three other children with MMS in our series underwent successful stem cell transplantation from HLA-matched siblings.

Stroke mortality

Two children (8%) aged four and eight years died shortly after arrival at our hospital; both had recurrent strokes and never received chronic transfusion therapy before arrival, consistent with what was reported in Jeddah [24]. To improve the outcome, it is important to provide an easy access to the TCD and MRI/MRA facilities in all areas where children with SCD are at risk to develop stroke. In addition, early bypass surgery and bone marrow transplantation should be considered for these patients.

Limitations of the study

While this is a retrospective study, it is subject to several limitations. The first is being retrospective with a lack of valuable data, such as possible MRA scans before the first stroke episode. The primary impediment to the generalization of our results is the small sample size at one center, which makes it difficult to perform statistical tests and identify significant relationships in the data. In addition, the scope of our analysis and comparison of our data with data from other centers was limited by the lack of previous similar studies in Saudi Arabia.

Directions for future research

More studies, prospective and multicenter, are needed to determine the extent of the moyamoya problem in children with SCD in Saudi Arabia. These may help modify guidelines regarding performing MRA in children with SCD at a younger age in areas without routine Doppler ultrasound. Also, more specific genetic studies may help define the high-risk patients.

## Conclusions

In this study, the prevalence of overt stroke is 9% in children with SCD originating from the southwestern region of Saudi Arabia (26/286), and 61.5% of them (16/26) had MMS. It is absent in the children of eastern origin (99 children). In places where TCD facility is not available, further studies are required to determine if MRA brain screening of children with SCD may detect MMS before the onset of stroke and help initiate protective therapy.
